# Improving cognitive control in adolescents with post-traumatic stress disorder (PTSD)

**DOI:** 10.1016/j.brat.2017.03.017

**Published:** 2017-06

**Authors:** Susanne Schweizer, Zobair Samimi, Jafar Hasani, Alireza Moradi, Fatemeh Mirdoraghi, Mohammad Khaleghi

**Affiliations:** a Medical Research Council Cognition and Brain Sciences Unit, Cambridge, United Kingdom; b Educational Psychology Department, Faculty of Education & Psychology, Azarbaijan Shahid Madani University, Tabriz, Iran; c Clinical Psychology Department, Faculty of Education & Psychology, Kharazmi University, Tehran, Iran; d Psychology Department, Faculty of Education & Psychology, Ferdowsi University of Mashhad, Mashhad, Iran

**Keywords:** Posttraumatic stress disorder (PTSD), Cognitive control, Emotion regulation, Adolescence, Emotion, Affective working memory, Cognitive control training

## Abstract

The adverse impact of posttraumatic stress disorder (PTSD) on the developing mind in adolescence can extend well into adulthood. The developmental malleability of cognitive control capacity in this age group, however, may hold particular promise for cognitive training interventions. The present study investigated the effects of affective working memory (aWMT) compared to placebo-training on cognitive and affective functioning in adolescents with PTSD. 30 treatment-seeking adolescents trained for 20 days on either an affective dual *n*-back task (aWMT; *n* = 15) or a feature match task (placebo; *n* = 15). The aWMT group showed greater pre-to post-training increases in cognitive control as measured by the GoNogo task as well as improvements in symptoms of PTSD and increased use of adaptive emotion regulation strategies. These preliminary findings are promising given the potential for free and easy dissemination of the aWMT in schools and online.

## Highlights

• Cognitive control can be improved in adolescents with PTSD.
• Symptoms of PTSD are reduced following affective working memory training.
• Self-reported use of adaptive emotion regulation strategies can be increased.

## 1 Training cognitive control in adolescents with posttraumatic stress disorder

Adolescence is a time of heightened risk for exposure to traumatic events (Hanson et al., 2008) and subsequent posttraumatic stress disorder (PTSD; Kilpatrick et al., 2003, Merikangas et al., 2010). The impact of PTSD on the mind at one of the peaks in cognitive and affective development (Steinberg, 2005) is arguably particularly devastating as it is associated with impairment in a host of critical cognitive functions including memory and attention (for a review see: Hayes, VanElzakker, & Shin, 2012). A cognitive domain that may be particularly vulnerable to the effects of adolescent PTSD is executive functioning, specifically cognitive control. Cognitive control refers to individuals' capacity to select and attend towards goal-relevant information, while inhibiting attention and responses to irrelevant stimuli (Koechlin, Ody, & Kouneiher, 2003). Its underlying neural substrates in the frontoparietal control network undergo rapid development during adolescence and well into early adulthood (Blakemore & Choudhury, 2006). Previous research has shown cognitive control to be significantly impaired in PTSD with concurrent changes in this same frontoparietal control network (Aupperle, Melrose, Stein, & Paulus, 2012). While the etiological mechanisms through which cognitive control may be associated with the development and/or maintenance of symptoms of PTSD remain to be explored (Aupperle et al., 2012, Casey et al., 2005), potential pathways include (cf., Aupperle et al., 2012): inhibiting attention and responses toward potential triggers of unwanted memories (Catarino, Küpper, Werner-Seidler, Dalgleish, & Anderson, 2015); difficulties in disengaging attention from trauma-related stimuli (Pineles, Shipherd, Mostoufi, Abramovitz, & Yovel, 2009); and inability to inhibit maladaptive emotion regulation strategies (e.g., avoidance) in favor of more adaptive strategies (e.g., reappraisal; Aldao, Nolen-Hoeksema, & Schweizer, 2010). Critically, these putative mechanisms involve difficulties in the application of cognitive control in affective contexts. Any interventions aimed at enhancing cognitive control in PTSD should therefore do so in affective contexts. Data from the adult literature shows psychological interventions for PTSD to be successful at treating symptoms of posttraumatic stress disorders (Lee et al., 2016, Watts et al., 2013). Notably, however, these treatments place considerable demands on cognitive control over affective information (e.g., restructuring a trauma memory) and require patients to override pre-potent emotion regulatory responses to engage in more adaptive strategies. Increasing cognitive control in adolescents suffering from PTSD may therefore further augment, or speed up the treatment success of psychological interventions relying heavily on cognitive capacity.

In the current study we aimed to improve cognitive control in adolescents suffering from PTSD using an affective working memory training (aWMT), which relies heavily on applying cognitive control in affective contexts (Engen and Kanske, 2013, Schweizer and Dalgleish, 2013). Specifically, we used a culturally and developmentally modified version of the aWMT protocol from Schweizer et al., 2013, Schweizer et al., 2011, which has been shown to augment cognitive control over affective information at both automatic (measured with an emotional Stroop task) and volitional (measured with a film-based emotion regulation task) levels of processing in healthy adults. Furthermore, at the neural level of analysis participants receiving aWMT showed optimized recruitment of the frontoparietal control network during emotion regulation after the training intervention, compared to a sham training group (Schweizer et al., 2013). The aWMT is a dual *n*-back task that requires participants to update and maintain visual (faces with affective expressions) and auditory (affectively-laden words) information. Participants have to indicate whether the face they are currently seeing is in the same spatial location as the one presented *n* positions back and/or whether the word they are hearing is the same as the one heard *n* trials ago (*n* is always titrated at participants' optimal performance level). Critically, the affective expression of the faces must be ignored, while the content of the words must be attended to for optimal task performance. Previous research has shown that early life trauma is associated with altered processing of negative facial expressions (i.e., increased attentional biases toward negative information and increased amygdala activation), especially threatening information such as angry faces (e.g., Dannlowski et al., 2012). Ignoring the affective content of these faces might therefore be especially challenging to this adolescent sample with PTSD. More generally, PTSD has been associated with altered emotional processing of a wide range of affective stimuli including emotional scripts, faces, words, and images (for a review see: Etkin & Wager, 2007) with patients showing prepotent attentional orienteering biases toward negative information (Bar-Haim et al., 2007, El Khoury-Malhame et al., 2011). The challenges posed by the task for the adolescents with PTSD might then be two-fold: first, inhibiting attention toward the affective content of the facial stimuli whilst recording their spatial location and second, to attend to and disengage from the negative words as appropriate to continuously successfully update them in WM.

We hypothesized that adolescents suffering from PTSD: would be able to train on the aWMT (H1) and that aWMT will lead to greater transferable gains in cognitive control, as measured on an untrained GoNogo task (Ghaderi, Khodadadi, & Abbasi, 2010), compared to a placebo-training (H2). Adolescents who trained on the aWMT were further predicted to report: engaging in more adaptive and fewer maladaptive emotion regulation strategies (H3) and reduced levels of posttraumatic stress symptoms (H4) compared to those who trained on the placebo-task. Finally, improved emotion regulation and reduced posttraumatic stress symptoms were hypothesized to change as a function of improved cognitive control (H5).

## 2 Methods

### 2.1 Participants

30 adolescents (20 female; age average 15.43, age range, 14 to 18) were recruited from the Khane Mehr Center, Karaj, Iran. To be included participants needed to have a current diagnosis of PTSD and not meet full criteria for any other axis I or II disorder as assessed with the Farsi version of the structured clinical interview for DSM-IV (SCID; First et al., 1995, Shooshtari et al., 2007). All participants were awaiting but not currently in treatment for their posttraumatic stress symptoms. Following inclusion participants were randomly allocated to either the aWMT (*n* = 15, 9 female) or a placebo training group (*n* = 15, 11 female). The sample size was included to have 95% power to detect a between-within subject interaction for the moderate to large effect on emotion regulation observed in our previous work in healthy subjects (Schweizer et al., 2013) and to account for a potential attrition rate of 0.30.

### 2.2 Training tasks

*Affective Working Memory.* The aWMT (adapted version of the task used by Schweizer, Hampshire, & Dalgleish, 2011) comprised an affective dual *n*-back task consisting of a series of trials each of which involved simultaneous presentation of a face (500 ms) on a 4 × 4 grid on a computer screen and a word (500 ms) over headphones. Each picture-word pair was followed by a 250 ms interval during which participants responded via a button press to indicate whether either or both stimuli matched the stimuli presented *n-*trials back. Words (e.g., death, rape) and faces were negatively valenced (fearful, angry, sad). The task always started at *n* = 1 and *n* increased by one when participants detected 60% or more of the targets accurately or reduced by one if the participants responded to fewer than 20% of the target trials correctly. The task included two types of feedback: for auditory targets, participants heard an unpleasant tone for missed targets and a pleasant note if the target was identified accurately. For correctly identified visuospatial targets a green happy smiley appeared and a red sad smiley for missed targets.

*Placebo training.* In the placebo task participants saw two panels with geometrical shapes on a computer screen. The top panel showed three target shapes, which the participants had to identify with a mouse click in the bottom panel. These panels included 5–13 distractor shapes in addition to the targets. The number of distractors presented together with the targets was random (i.e., not titrated to performance).

### 2.3 Questionnaire measures

*Impact of Event Scale-Revised.* This well-validated 22-item scale assesses symptoms of avoidance, intrusions, and arousal in three subscales (Creamer, Bell, & Failla, 2003). The Farsi version of the scale has shown good psychometric properties (Panaghi & Mogadam, 2006).

*Cognitive Emotion Regulation Questionnaire (CERQ) – child version.* The CERQ assesses adaptive (acceptance, positive refocusing, refocus on planning, positive reappraisal and putting into perspective) and maladaptive (self-blame, other-blame, rumination and catastrophizing) emotion regulation strategies (Garnefski, Kraaij, & Spinhoven, 2001). The Farsi version's psychometric properties are satisfactory (e.g., Hasani, 2010).

### 2.4 Transfer measure

*Cognitive control.* The GoNogo task was administered to measure cognitive control (Hester, Fassbender, & Garavan, 2004). On this task cognitive control is required to inhibit prepotent motor-responses to infrequent NoGo trials, in the context of response readiness to frequent Go trials (Hester et al., 2004, Verbruggen and Logan, 2008). In the current version of the task participant were presented with geometrical shapes for 500 ms, where a triangle-like shape was the target shape and responses (key press) had to be inhibited to all other geometrical shapes (Ghaderi et al., 2010). There were 100 trials (70% Go-trials). Three separate measures were derived from the task: commission errors (keypress for non-targets), inappropriate inhibition (omission errors, no keypress for targets) and reaction time on correct trials.

### 2.5 Procedure

Prior to the study, informed consent was obtained from the participants, their guardians and school officials. At a pre-training assessment session, participants completed the set of questionnaires as well as the Go-Nogo task. The study was a single blind-randomized design where the experimenters who were present in the classroom, were aware of participants training-group randomization. Participants were aware that one of the training tasks was a placebo task, but they were not informed, which condition they were randomized to, nor their guardians and teachers. The session was completed by explaining the training task to the participants. Participants then completed 20 sessions of the aWMT lasting between 30 and 45 min (dependent on level of *n*–back achieved) at the school on weekdays. The placebo training group completed 20 sessions of their training task for a fixed duration of 30 min. At the post-training session, which included all the measures administered at pre-training, participants were debriefed and compensated with an aquatic center gift card. All computer-based testing and training was done in groups, on a 15 inch laptop and under supervision of a post-graduate psychology student.

## 3 Results

### 3.1 Participant characteristics

The groups did not significantly differ in demographic characteristics including age, education, gender and socioeconomic status (Table 1). The placebo group did, however, report engaging in more adaptive and maladaptive emotion regulation strategies and made more errors on the GoNogo task at pre-training (Table 1).

### 3.2 Training effects

In line with our first hypothesis adolescents were able to train on the aWMT. aWMT performance showed a significant linear increase across the 20 days of training, *F* (1, 14) = 55.80; *p* ≤ 0.001, *η*_*p*_^*2*^ = 0.80, with average levels of *n* achieved increasing from *M* = 1.13, *SD* = 0.35, range = 1–2 at the start of training to *M* = 3.40, *SD* = 0.83, range = 2–5 on the 20th training day. Given the issues surrounding significance testing we additionally report Bayesian statistics for all^1^ our results (Rouder, Speckman, Sun, Morey, & Iverson, 2009). Specifically, we performed Bayesian analysis of variance which result in a Bayes factor (BF_10_) that represents the likelihood of the experimental hypothesis being true given the null hypothesis. All Bayesian analyses were computed using JASP (JASP Team, 2016), with the exception of Bayes factors for multivariate analyses, which were computed based on the formula: Bayes factor in support of the experimental hypothesis (BF_10_) = *n* x ln (*SS*_*error*_/*SS*_*total*_) + *k*_*H1*_ + ln(*n*), whereby *k* is amount of free parameters in experimental hypothesis (e.g., number of conditions).

For H1 Bayesian analyses confirmed our conclusions based on significance testing with BF_10_ > 100. A Bayes factor exceeding 100 is considered conclusive evidence in favor of the experimental hypothesis (Jeffreys, 1998).

### 3.3 Transfer effects

**Fig. 1:**
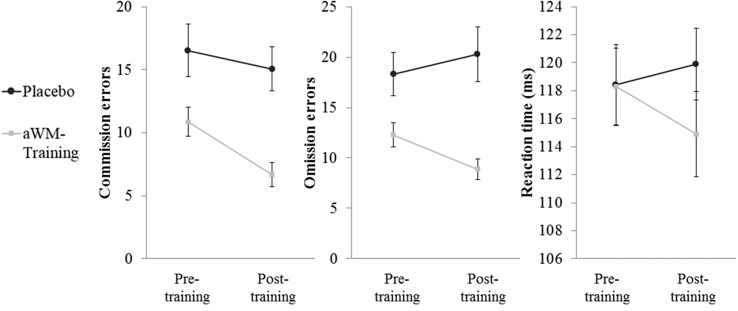
Pre-to post-training changes in cognitive control performance across groups.

*Cognitive control.* A multivariate mixed between-within-subject analysis of variance (MANOVA) was used to assess the second hypothesis that aWMT would improve cognitive control as measured by the GoNogo-task. We used a multivariate design because we did not have specific predictions regarding the tasks separate outcome measures (commission errors, omission errors and reaction time). The results revealed an interaction between time (pre-, post-training) and training group (aWMT, placebo), Wilk's λ = 0.71, *F* (3, 26) = 3.61; *p* = 0.03, *η*_*p*_^*2*^ = 0.29, BF_10_ > 100; Fig. 1. Deconstructing this interaction using univariate comparisons revealed that, compared to the placebo group the aWMT group showed a greater reduction in commission, *F* (1, 28) = 3.08; *p* = 0.045, *η*_*p*_^*2*^ = 0.10, BF_10_ = 0.95 and omission errors, *F* (1, 28) = 6.69; *p* = 0.01, *η*_*p*_^*2*^ = 0.19, BF_10_ = 3.55 from pre-to post-training. There were no significant differences in pre-to post-training reaction times, *F* (1, 28) = 2.31; *p* = 0.07, *η*_*p*_^*2*^ = 0.08, BF_10_ = 0.87 between the groups. Bayesian analysis provided moderate support for the significant time-by-group interaction for errors of omission, but did not support a group-by-time interaction for commission errors or reaction time.

Finally, given the significant group-by-time interactions for commission and omission errors we looked at the effect of time in each group separately for these two outcomes. The aWMT group showed a significant reduction in both commission and omission errors over time *F* (1, 14) = 61.33; *p* ≤ 0.001, *η*_*p*_^*2*^ = 0.81, BF_10_ > 100 and *F* (1, 14) = 5.17; *p* = 0.04, *η*_*p*_^*2*^ = 0.27, BF_10_ = 3.20, respectively. In contrast, the placebo group showed non-significant changes for both commission and omission errors, *F* (1, 14) = 1.00; *p* = 0.33, *η*_*p*_^*2*^ = 0.07, BF_10_ = 0.48 and *F* (1, 14) = 1.86; *p* = 0.20, *η*_*p*_^*2*^ = 0.12, BF_10_ = 0.64, respectively. Here the Bayesian analyses provided conclusive (commission errors) and moderate (omission errors) support in favor of an effect of time in the aWMT group, and did not support an effect of time in the placebo group.

*Emotion regulation.* The third hypothesis predicted that aWMT would lead to the engagement of more positive and fewer negative emotion regulation strategies. We had no predictions about specific emotion regulation strategies and therefore computed separate adaptive strategies and maladaptive strategies sum scores. These two scores were entered into a mixed between-within-subject MANOVA, which showed a significant time-by-group interaction when comparing changes in emotion regulation strategies reported from pre-to post-training, *F* (2, 27) = 16.61; *p* < 0.001; *η*_*p*_^*2*^ = 0.55, BF_10_ > 100; Table 2. Subsequent univariate analyses revealed a significant group-by-time interaction in the use of adaptive strategies, *F* (1, 28) = 19.11; *p* < 0.001; *η*_*p*_^*2*^ = 0.41, BF_10_ > 100, but not in the use of maladaptive strategies across groups, *F* (1, 28) = 0.87; *p* = 0.36; *η*_*p*_^*2*^ = 0.03, BF_10_ = 0.54.

To investigate the significant group-by-time interaction for positive emotion regulation strategies further, we analyzed the effect of time on adaptive emotion regulation in each group separately. The aWMT group showed a significant increase in the use of adaptive strategies across time, *F* (1, 14) = 68.50; *p* < 0.001; *η*_*p*_^*2*^ = 0.83, BF_10_ > 100, but not the placebo-training group, *F* (1, 14) = 1.00; *p* = 0.33; *η*_*p*_^*2*^ = 0.07, BF_10_ = 0.49. In contrast with our fifth hypothesis, training-related increases in the use of adaptive emotion regulations were not significantly associated with improvements in cognitive control^*2*^ in the overall sample, *β* = −0.28, 95% CI [–1.26, 0.17], *t* = 1.56, *p* = 0.13, *R*^*2*^ = 0.08 nor in each group separately, *p's* > 0.32.

*Symptoms of posttraumatic stress.* Finally, in line with the fourth prediction, that aWMT would lead to a greater (relative to placebo-training) reduction in PTSD symptoms, there was a significant group-by-time interaction, *F* (1, 28) = 53.00; *p* < 0.001; *η*_*p*_^*2*^ = 0.65, BF_10_ > 100. Univariate analyses revealed a significant reduction in posttraumatic stress symptoms in the aWMT group (pre-training IES-R = 49.60, *SD* = 5.15; post-training = 40.93, *SD* = 6.34), *F* (1, 14) = 87.96; *p* < 0.001; *η*_*p*_^*2*^ = 0.86, BF_10_ > 100, but not the placebo-training group (pre-training IES-R = 50.40, *SD* = 3.85; post-training = 49.53, *SD* = 4.22), *F* (1, 14) = 2.55; *p* = 0.13; *η*_*p*_^*2*^ = 0.15, BF_10_ = 0.80. In line with our fifth hypothesis the reduction in symptoms of PTSD was predicted by training-related changes in cognitive control^2^ in the overall sample, *β* = 0.40, 95% CI [0.04, 0.60], *t* = 2.31, *p* = 0.03, *R*^*2*^ = 0.16. When investigating the association in each group separately the association was no longer significant, however, interestingly the groups showed opposite association patterns with the aWMT showing a positive association between training related gains in cognitive control and a reduction in symptoms of PTSD, *r* (15) = 0.36 whereas the placebo group showed an inverse association between training-related changes in cognitive control and self-reported PTSD, *r* (15) = 0.41.

## 4 Discussion

The primary aim of the present study was to investigate the possibility of improving cognitive control in adolescents suffering from PTSD using an aWMT task. Compared to 20 sessions of non-WM-dependent placebo-training, the aWMT led to greater pre-to post-training gains in cognitive control as measured by performance on a GoNogo task (omission errors), greater increase in the use of adaptive emotion regulation strategies and a greater decrease in symptoms of posttraumatic stress. Moreover, overall reductions in PTSD symptoms were associated with greater training-related gains in cognitive control, however, greater use of adaptive strategies was unrelated to increased cognitive control at post-training.

In line with our hypothesis participants improved linearly on the aWMT task across training. Their post-training performance (*M* = 3.40, *SD* = 0.83) matched healthy adults' capacity at pre-training (range: *M* = 2.6 (*SD* = 0.83) – *M* = 4.5 (*SD* = 1.07); Schweizer et al., 2013, Schweizer et al., 2011). The finding that aWM and cognitive control as measured by omission errors on the GoNogo task (though not commission errors and reaction time) can be improved in this patient group is particularly promising given previous work showing reduced cognitive control over affective material in individuals with PSTD (Schweizer and Dalgleish, 2011, Schweizer and Dalgleish, 2016). Using a delayed-match-to-sample task, which presented participants with negative distractors during the delay between encoding and retrieval, Morey, Petty, Cooper, LaBar, and McCarthy (2008) showed that reduced affective WM performance in PTSD was associated with differential activation in the ventrolateral PFC compared to trauma-exposed controls without PTSD. This structure shows significant development during adolescence and its maturation is associated with improvements in cognitive control (Casey et al., 2000, Luna et al., 2010). Moreover, the vlPFC has been reliably implicated in inhibition (for a review see: Aron, Robbins, & Poldrack, 2004), the component of the GoNogo, for which the aWMT showed the most robust improvement. The promising results from the present study, then may be partially accounted for by the particular developmental malleability of the neural substrates involved in the cognitive control of emotions in adolescence. Future research should clarify firstly, whether the training gains observed in the present study are (if replicated) indeed associated with changes in the neural substrates of affective WM. Second, support should be sought for the developmental sensitivity of adolescents to cognitive training interventions by comparing them to other age groups (e.g., adults). Prospective research should further assess whether over time the gains in cognitive control observed in the aWMT relative to the placebo group will be potentiated, arguably by optimizing the engagement of task-relevant neural substrates.

Particularly, encouraging was the finding that aWMT was not only associated with gains in cognitive control but also augmented use of adaptive emotion regulation strategies and greater reduction in posttraumatic stress symptoms. Schweizer et al. (2013) previously showed that following aWMT psychologically healthy individuals were more successful at downregulating distress employing an adaptive emotion regulation strategy (i.e, reappraisal; *cf* Buhle et al., 2014). However, it should be noted this finding is in contrast with previous studies showing cognitive control training to lead to reduced report of maladaptive (ruminative brooding) but not increased adaptive strategies (e.g., Hoorelbeke et al., 2016, Siegle et al., 2014). A potential account for these discrepant findings is that the aWMT requires individuals to exert cognitive control over affective stimuli. Arguably the affective stimuli make this aWMT a closer approximation to the deployment of cognitive control in the presence of affective information (endo- and exogenous) encountered in everyday life – compared to a “cold” neuropsychological training task such as the adaptive Paced Auditory Serial Attention Task used in the studies showing no transfer to adaptive emotion regulation (Hoorelbeke et al., 2016; Siegle et al., 2014). However, the current study did not include a neutral WMT control condition. Consequently, it is possible that training-related gains were a function of WMT with the affective component having no impact on any of the observed transfer effects (Becker et al., 2015, Keshavan et al., 2014). In contrast, with this conclusion is our own previous work in healthy individuals, which showed training-related improvements on an affective Stroop task only in an aWMT group but not in a group that trained on a neutral version (i.e., including only neutral stimuli) version of the same WMT paradigm (Schweizer et al., 2011).

The results from the current study need to be interpreted with caution, however, given the small sample size. Significant effects in small samples are likely to represent an overestimation of the effect in the population (Button et al., 2013). Similarly, promising results have been observed for other computerized training interventions when small sample sizes were included (Amir, Beard, Burns, & Bomyea, 2009) but later failed to reliably replicate (Mogoaşe, David, & Koster, 2008). It is therefore critical to replicate the current findings in future research, ideally conducted by a different research group. Secondly, the placebo-training task in the current study did not include any affective stimuli, which makes it possible that the transfer effects to posttraumatic stress symptoms and use of adaptive emotion regulation strategies were due to repeated exposure to negative stimuli (Foa, Chrestman, & Gilboa-Schechtman, 2008). To address this issue we have developed an analogue placebo task including the visual stimuli from the aWMT and our preliminary findings have shown this affective placebo task to have no effect on symptoms of depression nor cognitive control (Schweizer, Carbon-Lee, Auer, Hampshire, & Dalgleish, 2016), though of course it may still impact on symptoms of PTSD. Moreover, the findings on the cognitive control task (i.e., GoNogo) in the current study are unlikely to be accounted for by exposure effects as the GoNogo task did not include affective material. Another limitation of the study design is that the transfer tasks included neither a non-trained measure of WM, nor a behavioral measure of affective control such as the emotional Stroop task (Kahan & Hely, 2008). Transfer to these measures have, however, previously been shown using the aWMT in healthy adults (Schweizer et al., 2016, Schweizer et al., 2013, Schweizer et al., 2011). Furthermore, training-related changes in emotion regulation and symptoms of posttraumatic stress were assessed using self-report questionnaires, which are prone to demand effects. And while participants were blind to their treatment allocation both training conditions were administered in class, which makes it possible (even likely) that participants were aware of the intervention condition they were in. That is, one of the training tasks was clearly affective and the other was affect-neutral. Given the nature of their condition participants were likely to deduce which of the two training conditions was intended as the active condition. Moreover, the placebo group significantly differed from the aWMT group at pre-training, showing lower levels of cognitive control (more errors on the GoNogo task) and reporting using more adaptive and maladaptive emotion regulation strategies. This raises the possibility that the training might be more efficient in individuals who have a higher cognitive control capacity at baseline. Secondly, it is possible that the greater gains in adaptive emotion regulation strategies in the aWMT group were in part due to with the placebo group having less room for improvement. On a positive note, the design did support the feasibility of running these type of computerized training interventions in school settings. Inferences from the current sample are further limited by the fact that only participants with a “pure” diagnosis of PTSD were invited to the study, whereas research in adolescence has shown that 40% of individuals with an axis one disorder have a comorbid disorder (Merikangas et al., 2010). Training effects may be attenuated, or enhanced, in those with a comorbid diagnoses or may depend on the nature of the comorbidity. Finally, the current study included generic negative stimuli. Using trauma-specific stimuli may make the training task even more effective in terms of augmenting patients' cognitive control over unwanted trauma-related thoughts and feelings.

In conclusion, this study provides a proof of principle study that cognitive control in adolescents with PTSD can be improved using a computerized aWMT task. The aWMT led to reductions in self-reported symptoms of posttraumatic stress and increased recruitment of adaptive emotion regulation strategies. The aWMT carries potential promise as an adjunct to existent cognitively demanding PTSD treatment interventions such as trauma-focused cognitive behavior therapy (Cary & McMillen, 2012). A major asset of the aWMT is that it can be administered online for free anywhere in the world where people have access to the internet.

## Funding source

SS is supported by UK Medical Research Council Programme MC-A060-5PQ60.

## Conflict of interest

No competing interests.

## Figures and Tables

**Table 1:** Pre-training demographic, clinical and cognitive characteristics across training groups.

	aWMT	Placebo training	*t*	*χ*^2^	*p*
	*n* = 15	*n* = 15			
Demographic					
Age in years, *M* (*SD*)	15.40 (1.24)	15.46 (1.06)	0.15		0.87
Education in years, *M* (*SD*)	9.93 (0.79)	10.20 (0.86)	0.87		0.38
Female, *n*	9	11		0.60	0.43
SES, High:Middle:Low	0:15:0	0:15:0		–	–
Clinical					
PTSD symptoms, *M* (*SD*)	49.60 (5.15)	50.40 (3.85)	−0.48		0.63
Adaptive strategies, *M* (*SD*)	50.40 (9.46)	60.13 (9.33)	−2.84		0.01
Maladaptive strategies, *M* (*SD*)	38.67 (6.16)	44.07 (7.40)	−2.33		0.03
Cognitive					
Commission errors, *M* (*SD*)	10.87 (4.53)	16.53 (8.02)	−2.38		0.03
Omission errors, *M* (*SD*)	12.27 (4.65)	18.33 (8.34)	−2.46		0.02
Reaction time, *M* (*SD*)	118.33 (10.59)	118.40 (11.16)	−0.02		0.99

SES = Socioeconomic status based on family's annual household income.

**Table 2:** Pre-to post-training changes in affect and emotion regulation.

	Placebo		aWMT	
	Pre-training	Post-training	Pre-training	Post-training
	*M* (*SD*)	*M* (*SD*)	*M* (*SD*)	*M* (*SD*)
Adaptive strategies	60.13 (9.33)	57.07 (14.79)	50.40 (9.46)	62.27 (10.96)
Maladaptive strategies	44.07 (7.40)	30.73 (6.53)	38.67 (6.16)	22.93 (4.04)

Adaptive strategies = sum score of all the CERQ subscales assessing adaptive emotion regulations strategies (acceptance, positive refocusing, refocus on planning, positive reappraisal and putting into perspective); maladaptive strategies = sum score of all the CERQ subscales assessing maladaptive emotion regulation strategies (self-blame, other-blame, rumination and catastrophizing).

## Footnotes

1 The one exception were the mediation analyses used to investigate the fifth hypothesis for which we were not aware of a standard approach to computing Bayes factors.

2 We chose omission errors *post hoc* as they showed the most reliable pre-to post-training changes. However, the result pattern did not change if we used the *a priori* specified composite measure of cognitive control.
